# Effect of Immunosuppressive Drugs on the Changes of Serum Galactose-Deficient IgA1 in Patients with IgA Nephropathy

**DOI:** 10.1371/journal.pone.0166830

**Published:** 2016-12-08

**Authors:** Min Jeong Kim, Stefan Schaub, Karen Molyneux, Michael T. Koller, Susanne Stampf, Jonathan Barratt

**Affiliations:** 1 Clinic for Transplantationsimmunology & Nephrology, University Hospital Basel, Basel, Switzerland; 2 Department of Infection, Immunity & Inflammation, University of Leicester, Leicester, United Kingdom; 3 Basel Institute for Clinical Epidemiology and Biostatistics, University Hospital Basel, Basel, Switzerland; Peking University First Hospital, CHINA

## Abstract

Galactose-deficient IgA1 (Gd-IgA1) and IgA-IgG complexes are known to play an important role in the pathogenesis of IgA nephropathy (IgAN). We aimed therefore to determine the impact of immunosuppression on the serum levels of Gd-IgA1, total IgA1 and IgA-IgG complexes in IgAN patients. In a retrospective study, serum samples from IgAN patients collected before transplantation (t_0_) and at 3- and 6-month posttransplant (t_3_ & t_6_) were used to measure the levels of Gd-IgA1, total IgA1 and IgA-IgG complexes. The area under the curves (AUC) of immunosuppressants was calculated by the plot of plasma trough level or dosage of each immunosuppressant *versus* time and was interpreted as the extent of drug exposure. Thirty-six out of 64 IgAN patients, who underwent kidney transplantation between 2005 and 2012, were enrolled. From t_0_ to t_3_, serum Gd-IgA1 and total IgA1 decreased significantly (24.7 AU (18.6–36.1) to 17.2 (13.1–29.5) (p<0.0001); 4.1 mg/ml (3.6–5.1) to 3.4 (3.0–4.1) (p = 0.0005)), whereas IgA-IgG complexes remained similar. From t_3_ to t_6_, Gd-IgA1 and IgA-IgG complexes significantly increased (17.2 AU (13.1–29.5) to 23.9 (16.8–32.0) (p = 0.0143); OD 0.16 (0.06–0.31) to 0.26 (0.14–0.35) (p = 0.0242)), while total IgA1 remained similar. According to median regression analysis, AUC of prednisone t_0-6_ was significantly associated with the decrease of Gd-IgA1 t_0-6_ (P = 0.01) and IgA1 t_0-6_ (p = 0.002), whereas AUC of tacrolimus t_0-6_ was associated with the decrease of IgA1 t_0-6_ (p = 0.02). AUC of prednisone t_0-3_ was associated with the decrease of IgA-IgG complexes t_0_-_3_ (p = 0.0036). The association of AUC prednisone t_0-6_ with Gd-IgA1 t_0-6_ remained highly significant after adjustment for other immunosuppressants (p = 0.0036). Serum levels of Gd-IgA1, total IgA1 and IgA-IgG in patients with IgAN vary according to the changing degrees of immunosuppression. The exposure to prednisone most clearly influenced the serum levels of Gd-IgA1.

## Introduction

One of the most remarkable findings in understanding the pathogenesis of IgA nephropathy (IgAN) is that an excess of poorly galactosylated IgA1 is present both in the serum and in the glomerular immune deposits of patients with IgAN [1, 2]. IgA1 has a unique hinge region between the first and second constant-region domains of its heavy chain [3]. This segment undergoes co/post translational modification by the addition of up to six *O*-glycan chains [4]. These chains comprise *N*-acetylgalactosamine (GalNAc) in *O*-linkage with either serine or threonine residues and may be extended by connecting galactose to GalNAc and are completed by adding sialic acid to galactose, GalNAc, or both. Defective galactosylation can lead to self-polymerization of IgA1 and facilitate their deposition in the kidney. Galactose-deficient IgA1 (Gd-IgA1) can also induce an autoimmune response with the production of glycan-specific IgG or IgA autoantibodies [5–7]. Although the exact mechanism of mesangial IgA1 deposition is not yet satisfactorily defined, immune complexes composed of Gd-IgA1 and IgG or IgA autoantibodies trigger mesangial cell activation, proliferation and also apoptosis [8, 9]. Activated mesangial cells release various proinflammatory, proproliferative and profibrogenic mediators and the subsequent inflammation, cellular proliferation, and the synthesis of extracellular matrix lead to the progression of IgAN [10–15].

Many studies have so far demonstrated that the serum level of galactose-deficient IgA1 (Gd-IgA1) of IgAN patients is significantly higher than that of healthy or disease controls [2, 16, 17]. Among patients with IgAN, 2 studies recently showed that the serum level of Gd-IgA1 at the time of renal biopsy significantly correlates with disease progression [16, 18]. In another study, the higher level of Gd-IgA1 was associated with more severe histologic findings [19]. Berthoux et al. indicated that not only the level of Gd-IgA1, but also the serum levels of IgG and IgA autoantibodies strongly associate with the progression of IgAN [17]. A recent publication suggested that the serum Gd-IgA1 is predictive of IgAN recurrence in the kidney graft [20]. How the concentration of serum Gd-IgA1 changes under various immunosuppressants remains however unclear. We therefore investigated the longitudinal changes of serum Gd-IgA1 and also total IgA1 and IgA-IgG complexes in relation to immunosuppressant in serially collected serum samples from IgAN patients before and after kidney transplantation.

## Materials and Methods

### Study design

Single-centre observational study with prospective collection of existing data from patient charts and de-novo lab-processing of existing serum samples from a previous clinical study of patients treated with renal transplantation [21].

### Patients

All patients with IgAN as the aetiology for ESRD, aged 18 years or older, who had kidney transplantation at the University Hospital of Basel between January 2005 and October 2012 were included, if serum samples at 3 & 6 months post-transplant were available. Patients who were on immunosuppressive drug by the time of transplantation or patients with a secondary form of IgAN were excluded. All patients gave written informed consent for the use of their data and samples for research [21]. The study was approved by the Research Ethics Committee of Canton of Basel. None of the transplant donors were from a vulnerable population and all donors or next of kin provided written informed consent that was freely given.

### Immunosuppressive regimens

Initial immunosuppression was selected based on the presence/absence of donor-specific anti-HLA-antibodies (HLA-DSA). Recipients of an allograft without HLA-DSA (i.e. normal risk patient) received basiliximab (Simulect, Novartis Switzerland), and triple therapy either with tacrolimus (Tac; Prograf, Astellas), mycophenolate mofetil (MMF; CellCept, Roche, Switzerland) and prednisone, or Tac, MMF and sirolimus (SRL; Rapamune, Wyeth). Recipients of an allograft with HLA-DSA (i.e. high risk) received induction therapy consisting of a polyclonal anti-T-lymphocyte globulin (ATG; ATG-Fresenius, Fresenius Medical Care) and intravenous immunoglobulins (IvIg) and triple therapy with Tac-MMF-prednisone. The target trough level of tacrolimus was 10–12 ng/ml during the first month and 8–10 ng/ml until 3 months. If there was no graft rejection in the 3-month protocol biopsy, tacrolimus trough level was decreased to 6–8 ng/ml. The target trough level of SRL was 4–8 ng/ml until 3 months. The MMF daily dose was 2g, if the body weight was over 50 kg (1.5 g/d otherwise). In normal risk patients, prednisone was tapered slowly to 7.5 mg/d until the end of 3 months and withdrawn, if there was no graft rejection in the 3-month biopsy. In high risk transplant recipients, prednisone was tapered to 0.1 mg/kg bw and maintained at least for 1 year.

### AUC of immunosuppressive drugs

Trough levels of Tac, MMF and SRL were measured regularly at each follow-up visit, i.e. at least once a week during the first 3 months and then every 2 weeks until 6 months. The area under the curve (AUC) was used to estimate the area inscribed by the plot of plasma trough level or dosage of each drug *versus* time. The AUC was interpreted as the extent of exposure to drug, and the unit of quantification was defined as ng.h/ml for Tac, mg.h/l for MMF and mg.h for prednisone. The AUC of SRL was not analyzed, because SRL was stopped within 3 months in all 5 patients, who were treated initially with SRL.

### Serum samples and kidney biopsy

Serum sampling collection was done immediately before transplantation (t_0_) and 3 & 6 months post-transplant by the time of protocol kidney biopsy (t_3_ & t_6_). Samples were aliquoted and stored at -80°C until the time of assay. In addition to light microscopic examination, immunofluorescence staining, especially for IgA was performed in every kidney biopsy.

### Measurements of serum total IgA1, Gd-IgA1 and IgA-IgG complexes

Serum IgA1 were quantified by specific ELISA. In brief, 96-well immunoplates were coated with rabbit anti-human antibodies to IgA (DAKO A0262), followed by blocking step with 2% bovine serum albumin (BSA) in PBS. 50 μl aliquots of standard and test serum samples were applied to duplicate wells. Standard curves were set up on each plate, using NIBSC serum standard (cat. No. 67/099) ranging from 1000 ng/ml to 2.0 ng/ml for IgA and from 863 ng/ml to 1.7 ng/ml for IgA1, respectively. Serum samples were diluted in PBS at 1:20,000. After overnight incubation, secondary antibodies to human IgA1 (sheep anti-human IgA1 (Binding Site)) were added for 2 hours incubation. For the development, horseradish peroxidase-conjugated anti-sheep/goat immunoglobulin antibody (Binding Site) was first applied for 1.5 hours incubation, followed by OPD/H_2_O_2_. The results were read as absorbance at 492 nm.

Levels of Gd-IgA1 were measured by lectin-binding assay using GalNAc specific lectin from the Helix aspersa (HA) using previously described ELISA method [1]. HA recognizes terminal *O*-linked GalNAc, and samples with lower terminal galactosylation and sialylation show higher HA binding. Briefly, IgA was captured on 96-well immunoplates, coated overnight at 4°C with 10 μg/ml anti-human IgA antibody (DAKO A0262), washed, and blocked with 2% BSA in PBS. Serum samples, diluted 1:100 in PBS, were applied to the plates (50 μL/well), in duplicate, and incubated overnight at 4°C. At this dilution, all wells on the plate were completely saturated with IgA1, which was confirmed by IgA1-specific ELISA. This enables equivalent amount of IgA1 from each sample to be tested by the assay. Gd-IgA1 was detected by incubation for 90 minutes with biotinylated HA (Sigma L8764) followed by HRP-conjugated avidin (DY 998). The reaction was developed with OPD/H_2_O_2_ substrate and the results read as absorbance at 492 nm. To create an IgA1-HA binding standard curve, three patients with the highest IgA1-HA binding and three patients/healthy subjects with the lowest IgA1-HA binding from the Leicester IgA cohort were selected. Equal amounts of sera from the 3 high IgA1-HA binders were pooled to create high standard and equal amounts of sera from the 3 low IgA1-HA binders were pooled to create low standard. The high and low pooled sera standards were then serially combined to create 11 different standard serums with arbitrary units (AU) of IgA1-HA binding ranging from 110 to 10. All standards were diluted 1:100 in PBS before use. The results were expressed as arbitrary units (AU). The intra- and inter-assay variations were < 10%.

Serum IgA-IgG complexes were also quantified by specific ELISA. In brief, 96 well immunoplates were coated with 100μl/well of AffiniPure F(ab’)_2_ Fragment Goat anti-human serum IgA, α chain specific (Jackson-Immuno Research) at 10μg/mL as the capture antibody and were incubated at 4°C overnight, washed and blocked with 2% BSA for 1 hour at room temperature. The dilution factor of the samples was 1/500 in PBS. After washing, 50μl/well of HRP-conjugated Polyclonal Rabbit Anti-human IgG (DAKO) was applied at 1/2000 dilution with PBS and plate was left to incubate at room temperature for ninety minutes. Plates were developed with 50μl/well of OPD (DAKO) with 30% of H_2_O_2_ substrate solution. The reaction was stopped with 75μl/well with 1N H_2_SO_4_ and read at 492nm. The results were expressed as optical density (OD).

### Statistical analysis

We performed non-parametric linear quantile regression analysis to study the effect of AUC of the different immunosuppressive drugs on the changes of serum total IgA1, Gd-IgA1 and IgA-IgG complexes from baseline to the 3 and 6 month time points. Quantile regression is an evolving body of statistical methods for estimating and drawing inferences about conditional quantile functions. For the current analysis, we used median regression analysis (tau = 0.5) with estimates of the asymptotic covariance matrix. For descriptive purpose, continuous variables were reported as mean ± SD. Skewed continuous variables were presented as median with interquartile range (IQR). All hypothesis tests are 2-sided, and the significance level was set to 5%. Statistical analyses were performed using Prism 5.0 (GraphPad, Software, Inc., La Jolla CA) and proc mixed of SAS Version 9.2.

## Results

### Baseline clinical data

Between January 2005 and October 2012, 64 patients with biopsy-proven IgAN underwent kidney transplantation in our center. Serum samples collected immediately before transplantation (t_0_) and at 3- and 6-month posttransplant (t_3_ & t_6_) were available from 36 out of 64 patients and these patients were not on immunosuppression by the time of transplantation. Patient characteristics are shown in Table 1.

**Table 1:** Patient characteristics.

Characteristics	n = 36
Age	53.4 ± 15.5
Sex, female/male	5 / 31
Duration of disease until RRT, months	15.5 (0–415)
Duration of dialysis until transplantation, months	20 (0–72)
Pre-emptive transplantation	4
Living/ deceased donors	16 / 20
Transplantation sequence, 1^st^ / 2^nd^	33 / 3
Number of HLA matches	3.3 ± 1.9
Positive donor specific anti-HLA antibody	4

Note: RRT, renal replacement therapy

### Longitudinal changes of serum Gd-IgA1, IgA1 and IgA-IgG complexes

**Fig 1:**
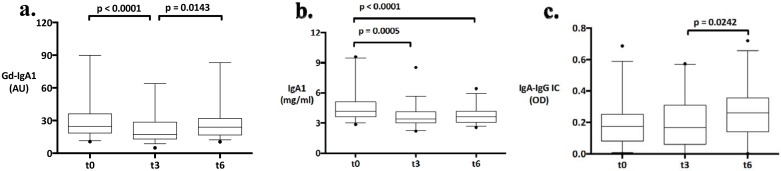
a, b, and c. Changes of galactose-deficient IgA1, total IgA1 and IgA-IgG complexes over time under influence of changing degrees of immunosuppression with tacrolimus, mycophenolate mofetil (MMF) and prednisone. Boxes represent the median 50% of patients, lower and upper box ends are the 25th and 75th percentiles, and the middle bar represents the median. Whiskers link the lower/ upper box ends with the 5th and the 95th percentile. Outliers are represented by a closed circle.

The serum levels of Gd-IgA1, IgA1 and IgA-IgG complexes were measured in longitudinally collected samples from t_0_, t_3_ & t_6_ (Fig 1a, 1b and 1c). From t_0_ to t_3_, serum Gd-IgA1 decreased significantly from 24.7 AU (median, 18.6–36.1 (IQR)) to 17.2 (13.1–29.5) (p<0.0001). From t_3_ to t_6_, however, the Gd-IgA1 significantly increased to 23.9 (16.8–32.0) at t_6_ (p = 0.0143). The serum IgA1 decreased also significantly from t_0_ to t_3_ (4.1 mg/ml (3.6–5.1) (t_0_) vs. 3.4 (3.0–4.1) (t_3_), p = 0.0005), remained however similar from t_3_ to t_6_ (3.4 (3.0–4.1) (t_3_) vs. 3.6 (3.0–4.2) (t_6_), p = 0.3388). The serum IgA-IgG complexes showed no significant change from t_0_ to t_3_ (OD 0.17 (0.08–0.25) (t_0_) vs. 0.16 (0.06–0.31) (t_3_), p = 0.6535), increased however significantly from t_3_ to t_6_ (0.16 (0.06–0.31) (t_3_) vs. 0.26 (0.14–0.35) (t_6_), p = 0.0242).

### Influence of various immunosuppressive drugs on the serum levels of Gd-IgA1, IgA1 and IgA-IgG complexes

All patients received induction therapy according to standard immunosuppression regimen: 4 patients with HLA-DSA received anti-thymocyte globulin and intravenous immunoglobulin; 32 patients received basiliximab. The initial immunosuppression in 31 patients consisted of Tac, MMF and prednisone and 5 patients received Tac, MMF and SRL. The area under the curves (AUC) of Tac, MMF and prednisone were calculated by trough levels or daily doses and shown in Table 2.

**Table 2:** Area under the curve of immunosuppressants.

**First 3 months (AUC t**_**0**_**-**_**3**_**)**	n = 36
tacrolimus (ng.h/ml)	136.7 ± 24.4
mycophenolate mofetil (mg.h/l)	42.6 ± 21.8
prednisone (mg.h)	175.8 ± 75.7
**4–6 month (AUC t**_**4-6**_**)**	
tacrolimus (ng.h/ml)	107.5 ± 23.8
mycophenolate mofetil (mg.h/l)	38.4 ± 24.4
prednisone (mg.h)	63.1 ± 67.3

Note: Daily dose of prednisone was calculated from only patients on the medication.

As scheduled, immunosuppression was reduced immediately after 3-month protocol kidney biopsy. Tac AUC t_4-6_ was significantly lower than Tac AUC t_0-3_ (p < 0.0001). Prednisone was withdrawn in 19 patients after 3-month kidney biopsy. Pred AUC t_4-6_ was also significantly lower than Pred AUC t_0-3_ (p < 0.0001). MMF AUC was not significantly different at both time periods. In 4 out of 5 patients with Tac/MMF/SRL, SRL was stopped within 3 months due to adverse events, such as leukopenia, polyoma virus nephropathy or SRL induced pneumopathy. One patient was switched to Tac/MMF/prednisone due to rejection in the 3-month biopsy.

**Fig 2:**
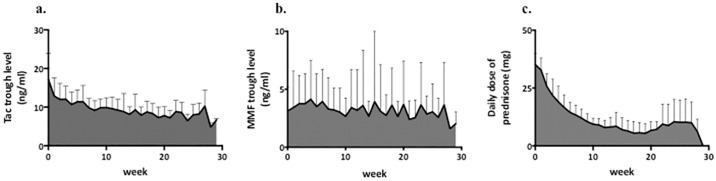
a, b, c Area under the curve of tacrolimus, MMF and prednisone over the study period from t_0_ to t_6_.

**Fig 3:**
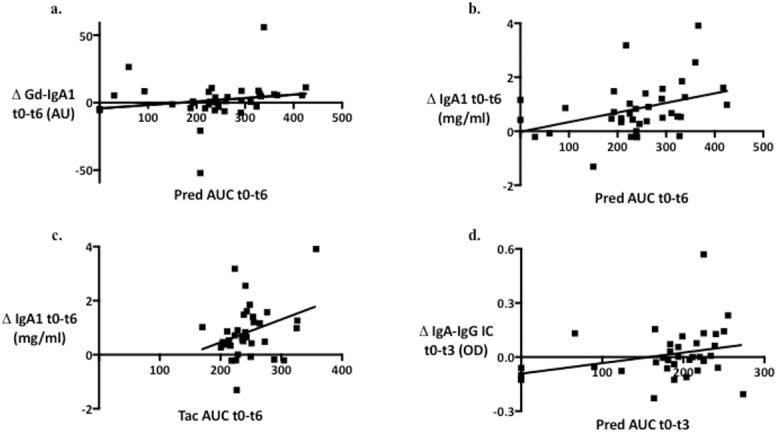
**Associations of a**. AUC t_0-6_ of prednisone and the degree of reduction of serum Gd-IgA1 between t_0_ and t_6_ (p = 0.01); **b.** AUC t_0-6_ of prednisone and the degree of reduction of serum total IgA1 between t_0_ and t_6_ (p = 0.0024); **c.** AUC t_0-6_ of tacrolimus and the degree of reduction of serum total IgA1 between t_0_ and t_6_ (p = 0.02)_;_ **d.** AUC t_0-3_ of prednisone and the degree of reduction of serum IgA-IgG complexes between t_0_ and t_3_ (p = 0.0036).

In order to examine the influence of different immunosuppressive drugs on the serum levels of Gd-IgA1, total IgA1 and IgA-IgG complexes, median regression analysis using AUC of different immunosuppressive drugs was performed (Figs 2 and 3). There was a significant association of the AUC of prednisone between t_0_ and t_6_ (AUC pred t_0-6_) with the decrease of the serum Gd-IgA1 between t_0_ and t_6_ (P = 0.01). In other words, an increase of 50 mg.h in Pred AUC would lead to a median decrease of 1.22 AU in Gd-IgA1, suggesting that a higher exposure to prednisone was associated with a stronger decrease in the serum Gd-IgA1. However, there was no significant association of AUC MMF t_0-6_ or AUC Tac t_0-6_ with the changes of Gd-IgA1 between t_0_ and t_6_. The decrease in the serum IgA1 between t_0_ and t_6_ was significantly associated with both AUC pred t_0-6_ (p = 0.002) and AUC Tac t_0-6_ (p = 0.02). In terms of IgA-IgG complexes, the decrease between t_0_ and t_3_ was significantly associated with AUC pred t_0-3_ (p = 0.0036).

We also performed multivariable adjusted median regression analysis for the post-transplant changes of serum Gd-IgA1, IgA1 and IgA-IgG complexes. The association of AUC pred t_0-6_ with the decrease in Gd-IgA1 between t_0_ and t_6_ remained significant after the adjustment for AUCs of MMF and Tac and ATG induction (p = 0.0036). The significant decrease in serum IgA1 levels in association with increasing AUC of pred and Tac t_0-6_ in the univariate analysis, however, disappeared in the multivariable model adjusted for other immunosuppressive drugs. The same was true for the changes of IgA-IgG complexes between t_0_ and t_3_.

### Recurrence of IgAN

During the median follow-up of 3.3 years (1.0–7.5), 5 patients (14%) developed recurrence of IgAN. Median time to recurrence was 6 months (3–12). Two patients showed microhematuria and one patient only proteinuria at the time of diagnosis. Two patients did not show any clinical findings of recurrence at the time of diagnosis, i.e. diagnosis by protocol biopsy. All other protocol biopsies at 3 and 6 months posttransplant from 31 patients were negative for IgA in the immunofluorescence staining. Median values of Gd-IgA1, IgA1 and IgA-IgG complexes at t_0_, t_3_ and t_6_ in patients with recurrence or non-recurrence were shown in Table 3. The levels of Gd-IgA1 were higher in the patients with recurrence at each time point, although the differences were not statistically significant (p = 0.07 at t0; p = 0.07 at t_3_; p = 0.28 at t_6_).

**Table 3:** Gd-IgA1, total IgA1 and IgA-IgG complexes in recurrent and non-recurrent patients at t0, t3 and t6.

	t0		t3		t6	
	recurrent	non-recurr	recurrent	non-recurr	recurrent	non-recurr
Gd-IgA1 (AU)	36.6 (27.4–66.6)	23.9 (17.4–32.9)	28.7 (20.3–41.5)	16.2 (12.9–28.2)	31.2 (21.6–40.9)	23.9 (16.0–31.7)
IgA1 (mg/ml)	4.1 (3.9–4.4)	4.3 (3.6–5.1)	3.2 (2.6–3.3)	3.6 (3.1–4.2)	2.8 (2.6–3.3)	3.7 (3.2–4.4)
IgA-IgG (OD)	0.26 (0.10–0.50)	0.16 (0.07–0.23)	0.10 (0.03–0.34)	0.18 (0.06–0.32)	0.27 (0.13–0.50)	0.26 (0.14–0.36)

## Discussion

In this study, we examined the impact of immunosuppression on the changes of the serum levels of Gd-IgA1, total IgA1 and IgA-IgG complexes in patients with IgA nephropathy using serum samples collected immediately before transplantation and 3 & 6 months posttransplant. While the serum levels of Gd-IgA1 and total IgA1 significantly decreased from t_0_ to t_3_, IgA-IgG complexes remained stable in this period. Under the scheduled reduction of immunosuppression between t_3_ and t_6_, the serum levels of Gd-IgA1 and IgA-IgG complexes increased significantly, whereas the serum levels of total IgA1 remained similar.

We therefore looked at, how various immunosuppressive drugs influence the serum levels of Gd-IgA1, total IgA1 and IgA-IgG complexes differently. According to median regression analysis, the decrease of serum Gd-IgA1 was significantly influenced by the degree of exposure to the prednisone therapy, whereas the decrease of total IgA1 by the degree of exposure to the prednisone or tacrolimus therapy. The decrease of IgA-IgG complexes seemed to be influenced by the prednisone therapy especially in the initial period (t_0_ to t_3_). In the multivariable adjusted regression analysis, however, only the association between the decrease of Gd-IgA1 t_0-6_ and the exposure to prednisone t_0-6_ was highly significant.

The initial decrease of Gd-IgA1 between t_0_ and t_3_ and the following increase between t_3_ and t_6_ may therefore be explained mainly by the decreasing exposure to prednisone after t_3_. Why the levels of Gd-IgA1 compared with that of total IgA1 or IgA-IgG complexes respond more sensitively to the degree of exposure to prednisone is not clear. The mechanism regulating IgA1 O-glycosylation is currently not well understood. Both *in vitro* and *in vivo* data suggest that T-cell cytokines, such as IL-4 and IL-5 stimulate the production of abnormally glycosylated IgA [22–24]. Regulation of such cytokines by immunosuppression might be one of the mechanisms involved in the variation of Gd-IgA1 level. It might also be that B-cell clones producing Gd-IgA1 are more susceptible to the changes of immunosuppression, especially to prednisone, than others.

The reduction of Gd-IgA1 between t_0_ and t_3_ cannot be explained simply by a reduction in total IgA1 due to the method of the analysis. During the lectin binding assay, all wells are completely saturated with IgA1. The detected difference of Gd-IgA1 concentration in each well therefore represents the true changes in Gd-IgA1 concentration. The increase of Gd-IgA1 between t_3_ and t_6_, despite the stable concentration of total IgA1, demonstrates the availability of the assay to detect the changes of Gd-IgA1 concentration.

Previous studies suggest that immunosuppression with steroids improve the long-term outcome of IgAN [25, 26]. The exact mechanisms by which steroids alter the course of IgAN are still unclear. Our question was whether immunosuppression could modify the components of multi-hit pathogenesis mechanism [27]. It remains however unclear, if the observed changes of the serum levels of Gd-IgA1, total IgA1 and IgA-IgG complexes under immunosuppression may have an impact on the pathogenesis and the clinical course of the disease. As an example, the presence of high levels of Gd-IgA1 in unaffected relatives of patients with both familial and sporadic IgAN suggests that additional factors are required for changes in IgA1 *O*-glycosylation to translate into clinical disease [28]. A recent randomized clinical trial by Rauen et al. also reported that immunosuppressive therapy in addition to ongoing supportive therapy was not beneficial in patients with IgAN with moderate proteinuria and chronic kidney disease stages 1 through 3 [29].

Although our study included patients with IgA nephropathy treated with renal transplantation, it is not possible to evaluate the association of serum levels of Gd-IgA1, total IgA1 and IgA-IgG complexes on the recurrence of IgAN due to the small number of patients, the low recurrence rate and the short follow-up period. Previous study by Coppo et al. showed no strong association between Gd-IgA1 level and recurrence of IgAN [30], however no clear statement can be drawn as sampling time was not clearly defined. On the other hand, a recent single center study by Berthelot et al. reported that the pre-transplantation serum Gd-IgA1 and IgA-IgG complexes were highly predictive for IgAN recurrence [20].

Our study has several limitations, such as small number of patients, inclusion of patients with kidney transplantation, observational manner and relatively short follow-up period. Our patients treated with kidney transplantation are however very valuable to examine the effect of immunosuppression on the serum levels of Gd-IgA1, total IgA1 and IgA-IgG complexes, as they are all exposed to immunosuppression following transplantation. During the post-transplantation course, immunosuppression is gradually reduced and this therefore provides a unique opportunity to observe the changes of those serum levels under changing degrees of immunosuppression.

## Conclusions

We demonstrate that the serum levels of Gd-IgA1, total IgA1 and IgA-IgG complexes in patients with IgA nephropathy vary according to the changing degree of immunosuppression. The exposure to prednisone therapy seems to influence the serum level of Gd-IgA1 most clearly. It needs to be investigated in large clinical studies of IgAN, whether these changes by immunosuppression influences the pathogenesis favourably, leading to an improvement of long-term clinical outcome, in both native kidney disease and recurrent disease.
